# TECHNOLOGY IN LONG-TERM CARE: DOES IT FACILITATE THE WORKFORCE OR IMPROVE SATISFACTION AND RETENTION?

**DOI:** 10.1093/geroni/igz038.2840

**Published:** 2019-11-08

**Authors:** Susan A Chapman, Joanne Spetz, Jacqueline Miller

**Affiliations:** University of California, San Francisco, San Francisco, California, United States

## Abstract

Emerging technological advances hold potential for individuals and caregivers to help an aging population in the home or long term care settings and has the potential to alter workforce needs and potentially mitigate the rising workforce demand. The purpose of the proposed research was to research available technologies that may facilitate, replace, or enhance recruitment, training, and retention of the LTC workforce. This qualitative study included environmental scan and literature search identifying 62 companies meeting criteria for potential impact on the workforce. Categories included wearables, robots, sensors/alerts, health/social data collection and tracking, family/caregiver communication services, online care management, online worker training, and facility or home health staffing systems. Thematic analysis findings of telephone interviews with 12 national and international companies included: 1) most development focused on software rather than hardware, 2) company founders generally had a tech background and start up experience along with personal family caregiving experiences, 3) data collected in the home often did not connect directly to the health care team, 4) payment was generally out of pocket to individuals or facilities under subscription services or contracts 5) worker retention improved when system allowed better client to worker matching, more control over shift scheduling, and more efficient staffing. There was little rigorous research on the impacts for care and services or which will have the greatest potential impact on the workforce providing direct care. Reimbursement from federal and private payers is minimal to date yet demand for government payment may grow.

